# Leveraging lessons learned from the COVID-19 pandemic for HIV

**DOI:** 10.1038/s43856-022-00175-8

**Published:** 2022-08-29

**Authors:** Thomas Calder, Tina Tong, Dale J. Hu, Jerome H. Kim, Karen L. Kotloff, Richard A. Koup, Mary A. Marovich, M. Juliana McElrath, Sarah W. Read, Merlin L. Robb, Philip O. Renzullo, M. Patricia D’Souza

**Affiliations:** 1 National Institute of Allergy and Infectious Diseases, National Institutes of Health, Bethesda, Maryland USA; 2 International Vaccine Institute, Seoul, Republic of Korea; 3 University of Maryland School of Medicine, Baltimore, MD USA; 4 Fred Hutchinson Cancer Research Center, Seattle, WA USA; 5 Walter Reed Army Institute of Research, Silver Spring, MD USA

**Keywords:** Viral infection, Medical research, SARS-CoV-2, HIV infections

## Abstract

The rapid development of COVID-19 vaccines and their deployment in less than a year is an unprecedented scientific, medical, and public health achievement. This rapid development leveraged knowledge from decades of HIV/AIDS research and advances. However, the search for an HIV vaccine that would contribute to a durable end to the HIV pandemic remains elusive. Here, we draw from the US government experience and highlight lessons learned from COVID-19 vaccine development, which include the importance of public-private partnerships, equitable inclusion of populations impacted by the infectious pathogen, and continued investment in basic research. We summarize key considerations for an accelerated and re-energized framework for developing a safe and efficacious HIV vaccine.

## Abstract (web summary)

Calder, Tong et al. discuss how the rapid development of COVID-19 vaccines benefited from HIV/AIDS research. They highlight lessons learned from the COVID-19 vaccine development experience that could accelerate and re-energize the development of a safe and efficacious HIV vaccine.

## Introduction

As we reflect on the monumental advances in treatment and prevention in response to the COVID-19 pandemic, we are now poised to synthesize and apply the experience gained to other global infectious disease challenges. Both HIV and COVID-19 emerged as new viruses without specific diagnostic tests, treatments, or prevention modalities; thus, initial containment relied on public compliance with behavior changes to mitigate spread. Both pathogens are RNA viruses with the propensity to mutate and diversify, making it challenging to develop interventions. Furthermore, the COVID-19 pandemic has highlighted the sustained threats to, and vulnerabilities of the public health system, including the impact of continued health inequities and marginalization of vulnerable communities. Much of the burden of both COVID-19 and HIV has been placed on vulnerable groups living in social conditions that make disease prevention difficult.

**Fig. 1:**
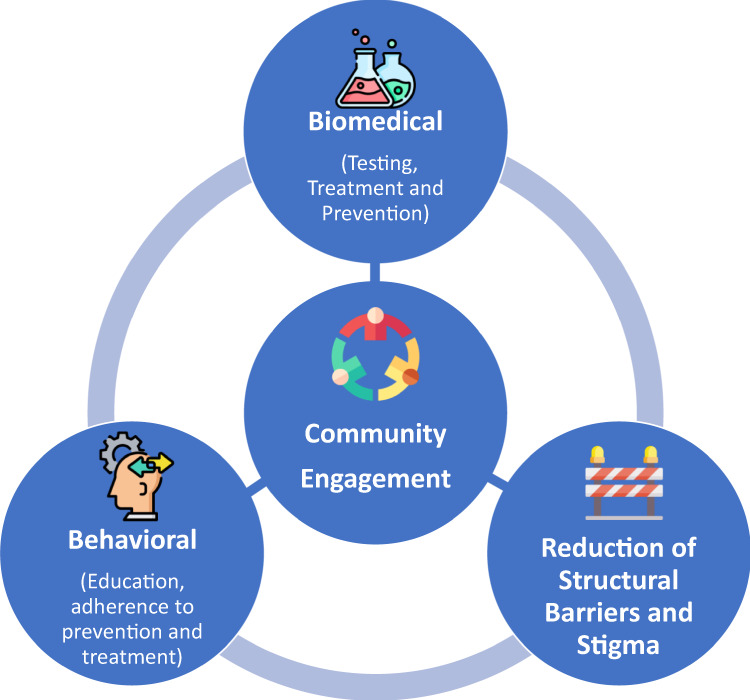
A combination prevention toolbox has applications both for HIV and COVID-19. For both HIV and COVID-19, a combination prevention toolbox has the potential to reduce the transmission and number of new infections to control the pandemics.

Over 40 years of research and investments in HIV vaccine research and development have shown that a multifaceted prevention approach should be comprehensive, but also data-driven. Similar to the prevention of COVID-19, HIV prevention priorities fall into three main categories: behavioral modification, biomedical innovation, and reduction of structural barriers. For both diseases, these prevention approaches have the potential to significantly reduce the transmission and hence the number of new infections, which are essential to control a pandemic (Fig. 1).

Recently, the Division of AIDS of the National Institute of Allergy and Infectious Diseases held a workshop entitled, “Leveraging the COVID-19 Experience to Accelerate the Development of a Safe and Efficacious HIV Vaccine” to assess how the COVID-19 experience can inform how to catalyze HIV vaccine development. Stemming from this workshop, several approaches were deemed most salient for accelerating HIV vaccine development: adapting basic and clinical science approaches; improving US government interagency collaborations and public-private partnerships; and reinvigorating the research community’s commitment to engaging and reaching diverse communities that are most impacted by the pandemic. This Perspective article focuses on the COVID-19 response initiated by the US government and how lessons learned can be applied to the HIV pandemic.

## HIV research advances provided a basis for COVID-19 research

Before we explore how the COVID-19 experience can be leveraged for HIV, we must first recognize that the successes and accelerated timeline of COVID-19 vaccine development were largely made possible due to decades of HIV research. Some of the major contributions include innovative approaches to rapidly identify and sequence new viral pathogens and new technologies for vaccine delivery, such as the nucleic-acid-based mRNA vaccines and adenovirus vector vaccines. Additionally, the creation of a stabilized COVID-19 spike protein  was, in part, possible due to years of investment in HIV structural biology research. The HIV vaccine field also provided a mature clinical trial infrastructure supported by the National Institutes of Health (NIH), which enabled immediate access to scientific experts and highly trained clinical staff. It also established the value of forging relationships between academia and industry and helped to strengthen health system capacities not only in the US but also globally by establishing an international footprint via clinical trial sites, particularly in resource-limited settings. Nevertheless, much more needs to be done to improve global access to vaccines and other preventive interventions by sharing intellectual property and enabling manufacturing and distribution equitably around the world^1^. Importantly, the many years of active community engagement in HIV research greatly informed COVID-19 vaccine developers and clinical site staff on how to reach and communicate with the most impacted and underserved communities.

HIV vaccine research is entering its fifth decade. A safe and effective HIV vaccine has been elusive, with significant challenges, but we are well positioned to adapt and learn from the COVID-19 vaccine experience. Only one HIV-1 vaccine efficacy trial undertaken to date, called RV144, has shown a modest reduction in HIV acquisition^2^. Evidence of protection against infection was not found in a subsequent trial, HVTN 702, of a conceptually similar vaccine design^3^. Similarly, no protection was observed in HVTN 705, a conceptually different vaccine^4^. In total, out of the seven HIV-1 vaccine efficacy trials successfully undertaken to date, six resulted in no efficacy. Other important approaches for HIV prevention include treatment to lower virus levels below that required for transmission; delivery of oral, injectable, or vaginal ring pre‐exposure prophylaxis with potent long‐acting antiretrovirals to prevent the virus from establishing infection; and passively delivered broadly neutralizing antibodies. However, these interventions require adherence, which is challenging in settings where stigma is prominent or where dependable access may be compromised due to logistics, cost, or other obstacles^5^. We believe the only guarantee of a sustained end of the AIDS pandemic lies in a combination of nonvaccine-based prevention methods and the development and deployment of a safe and sufficiently effective HIV vaccine.

## Sustained investment is required in basic science

The HIV and COVID-19 vaccine fields share a synergistic relationship, in which the HIV field has informed the development and testing of COVID-19 vaccines, leading to new advances that in turn may inform HIV vaccine design. This is evident in the use of structural biology for vaccine design. Many of the COVID-19 vaccines adopted a stabilized form of the spike protein that was based on sequential advancements from HIV, respiratory syncytial virus, and other coronaviruses, including severe acute respiratory syndrome-associated coronavirus (SARS-CoV) and Middle East respiratory syndrome-associated coronavirus (MERS-CoV)^6^. Given the successes of structure-based vaccine design, the HIV field could adapt lessons from COVID-19 and the many other viruses that encode a trimeric glycoprotein in the form of a type I fusion protein to mediate virus-cell fusion. However, the equivalent protein in HIV, the HIV Envelope (Env) protein poses additional unique challenges. These include the presence of a dense and poorly immunogenic Env glycan shield, molecular mimicry of Env epitopes that induce cross-reaction of antibodies with human proteins, and variable loop regions in Env that display epitopes of potent neutralizing antibodies, as well as non- or weakly neutralizing antibodies on a stabilized HIV Env spike^7,8^. Additionally, unmutated, or “germline” versions of broadly neutralizing antibodies (bnAbs) frequently do not bind to most Env proteins, requiring either the design of specific germline-targeting molecules or careful selection of immunogens to initiate a bnAb response^9^. Also, HIV-1 bnAbs have unusual traits such as autoreactivity, long third heavy chain complementary determining regions, and are enriched in rare somatic mutations, all of which make bnAb precursors either very rare owing to immune tolerance deletion or being difficult to activate. The study of the ontogeny of HIV neutralizing antibodies suggests that an effective vaccine will likely require multiple immunogens administered in a specific sequence to facilitate proper antibody development^10^. Further complexities unique to the HIV envelope include the global sequence diversity, integration of the viral genome into host cells, the long duration of a symptom-free period of infection, and the lack of spontaneous cures^11^. Thus, designing an HIV vaccine is a difficult problem that will require additional scientific discovery and continued virus strain surveillance, as immune responses to current circulating strains need to be triggered that are qualitatively different from natural infection.

A number of major contributions from the existing HIV Vaccine Trials Network (HVTN) were applied to the COVID-19 vaccine effort. For example, within the COVID clinical laboratory landscape, the network had existing validated immunology assays and quality assurance systems for HIV that met regulatory compliance for product licensure, which could be easily modified for COVID-19 vaccines^12,13^. The adaptation of these standardized binding assays to use SARS-CoV-2 proteins and neutralization assays with pseudoviruses or replication-competent viruses enabled comparisons across vaccine platforms and informed decisions on dose, regimens, and effectiveness of COVID vaccines based on antibody responses^14^. These same assays have also provided scientists with tools to quickly assess vaccine responses and potential effectiveness against the multiple variants of concern as they arose during the pandemic. While much of the focus of vaccine development and immune surveillance has been on the role of antibodies, less emphasis has been placed on understanding the role of T cells. Standardized assessment of vaccine-induced T-cell responses was largely limited to phase 1-2 trials because of the difficulty in collecting peripheral blood mononuclear cells from participants in the large-scale COVID-19 trials. However, mounting evidence suggests T-cell contributions to the host immune response are required for early, broad, and durable protection from SARS-CoV-2, especially in the setting of new variants of concern^15,16^.

Importantly, the use of standardized assays allow researchers to establish immune correlates of protection which allows data to be compared between studies where direct demonstration of effectiveness is difficult and time-consuming, such as when a vaccine is tested in new populations, or after dose modifications or manufacturing changes^17^. This approach could be relevant to the HIV vaccine field, as correlates of HIV-1 risk involving multiple immune responses have been identified from the RV144 HIV-1 vaccine efficacy trial^18^; however, these are not yet considered correlates of protection since they have not been confirmed in another vaccine efficacy trial. Finding a correlate of protection for HIV has been more challenging than it was for COVID-19. In the case of COVID-19, the hypothesis that antibodies against spike protein, whether elicited by natural infection or by vaccination are a correlate of protection against COVID-19 is supported by diverse lines of evidence^19^, including evidence supporting a mechanistic correlate of protection that can be derived from passive transfer of antibodies in experimental challenge studies and from studies of human monoclonal antibodies^20–22^.

For both HIV and SARS-CoV-2, more knowledge is needed about human immunology in tissue sites other than the blood. The SARS-CoV-2 virus does not replicate extensively in blood. It primarily infects epithelial cells on mucosal surfaces in nasal cavities, oral-pharyngeal spaces, and lungs, thus limiting contact with the systemic immune system^23^. Currently, the involvement of antigen-specific CD8 T cells, memory B cells, or secreted antibodies in those three sites in humans is poorly understood because specimens are difficult to obtain. Similarly, differential immune responses to HIV from the blood, gastrointestinal, genital tract secretions, and tissues have shown that mucosal responses are potentially relevant to the successful prevention of HIV-1 transmission through sexual exposure. Serum antibody levels and mucosal antibody levels following passive intravenous infusion of a broadly neutralizing anti-HIV antibody have been shown to be lower in the mucosa than serum, suggesting that the antibody potency required to protect against infection in tissue may be different from that measured in peripheral blood^24^. Thus, investigating blood as a surrogate for mucosal immune responses may pose a significant limitation as the level and the function of vaccine-elicited immune response and passively transferred antibodies in the mucosa may be incompletely represented. Moving forward, integrating mucosal tissue and secretion assessments into systemic assessments will provide a framework to develop next-generation vaccines that prevent viral infection.

SARS-CoV-2 viral dynamics and vaccine-induced protection vary by tissue compartment, with clinical consequences in the rates of protection for asymptomatic, mild/moderate infection versus severe disease and death. Immunity following natural infection with SARS-CoV-2, combined with vaccine-induced immunity, has not yet prevented the emergence and rapid spread of viral variants such as the highly transmissible delta (B.1.617.2) and omicron (B.1.1.529) variants. How long-lasting protective immunity can be achieved, and whether it can prevent the emergence of immune escape variants, remains unknown. A concerted research effort is needed to investigate the quality and durability of immune responses to SARS-CoV-2 and HIV in humans after both natural infection and systemically administered vaccines.

Another major lesson learned from the COVID-19 experience is that mRNA vaccines are safe and lead to a robust protective immune response against clinically symptomatic disease, but provide limited protection from infection. This technology offers several benefits for the creation of an HIV vaccine, but also presents several limitations that must be considered (Table 1). mRNA COVID vaccines have shown durability that can last months or possibly longer with boosting. Many infectious disease fields, including HIV, are now accelerating studies with this technology. For the HIV field, it offers the ability to quickly produce vaccines for several different HIV antigen configurations (e.g., complex tri-membrane stabilized trimers) that could be used to optimize regimen strategies, such as sequential immunization^25^. Given that developers can produce GMP material of mRNA vaccines quickly, and at a fraction of the cost of more traditional methods, this technology may expedite preclinical and clinical testing. Additionally, mRNA platform may enable iterative clinical trials to be used for HIV, in which the immunogen can be incrementally built, refined, and improved to generate the desired clinical responses.

**Table 1:** Benefits and limitations of HIV mRNA vaccines.

Benefits of mRNA vaccines	Limitations of mRNA vaccines
❖ Quick development timeline ❖ Affordable production ❖ Adjuvant not needed ❖ Protein purification not required ❖ Enables delivery of complex immunogens (e.g., membrane-bound trimers)	❖ mRNA instability ❖ Ultra-cold chain storage and transportation requirements ❖ Uncertainty of immunogenicity and durability

The mRNA vaccine technology presents several benefits and limitations that should be considered in designing future HIV mRNA vaccines.

Adjuvants are integral components of vaccines and play a critical role in augmenting the immune response. The COVID-19 experience has demonstrated the safety and effectiveness of novel adjuvants such as the TLR7/TLR8 agonist (used in COVAXIN®) and saponin adjuvants (used in Novavax-CoV2373). Given the rapid accumulation of safety data from clinical trials and subsequent deployment of these adjuvants for vaccines against COVID-19, these novel adjuvants can now be utilized for vaccines against HIV and other infectious pathogens. On a related note, encouraging safety and efficacy results were shown for the COVID-19 spike nanoparticle vaccine^26^, which was part of the Novavax vaccine and the only Emergency Use Authorization **(**EUA) nanoparticle platform for any vaccine to date. These nanoparticles have been shown to promote the formation and long-term persistence of germinal centers after vaccination. Nanoparticles may be particularly useful for the HIV vaccine field. The germline-targeting concept was validated in the International AIDS Vaccine Initiative G001 study, which used a self-assembling nanoparticle of engineered HIV Envelope proteins linked to a spherical protein structure, and demonstrated that the immunogen could activate naive B cells that produce precursors to a certain class of broadly neutralizing antibody^27^. As a next step, researchers at the NIH are engaging in public-private partnerships to develop and test several mRNA-based vaccines that harness the same approach to stimulate diverse naive B cells, including a vaccine regime with a germline-targeting prime with boost to induce broadly neutralizing antibodies against HIV (NCT05001373). In addition, an ongoing first-in-human study is evaluating whether delivery of soluble or membrane-bound HIV Env trimers via an mRNA platform is a promising platform for rapid and iterative HIV vaccine development (NCT05217641).

COVID-19 research is also making major strides in other aspects of vaccine research that could advance HIV vaccine development. The plethora of clinical and point-of-care testing products for COVID-19 have been crucial to track virus spread and monitor or identify the need for infection prevention and control measures, thus preventing transmission. The testing data could help guide effective clinical and public health policy and help improve study design efficiency when closely monitored during clinical trials. While the HIV field could benefit from more efficient testing technologies, there are key differences between HIV and SARS-CoV-2 that require the tests to be used in different ways. For example, vaccine-induced seropositivity (VISP) from an HIV vaccine does not equate to protection from infection. In fact, VISP adds an additional potentially stigmatizing challenge in that there is a need to distinguish the immune response resulting from vaccination versus infection, requiring an additional nucleic-acid test for confirmation of infection. This challenge, if unaddressed, can result in incorrect HIV diagnosis, consequent social harms, and inaccurate reporting to health agencies^28^.

## Government interagency collaborations and public-private partnerships

**Fig. 2:**
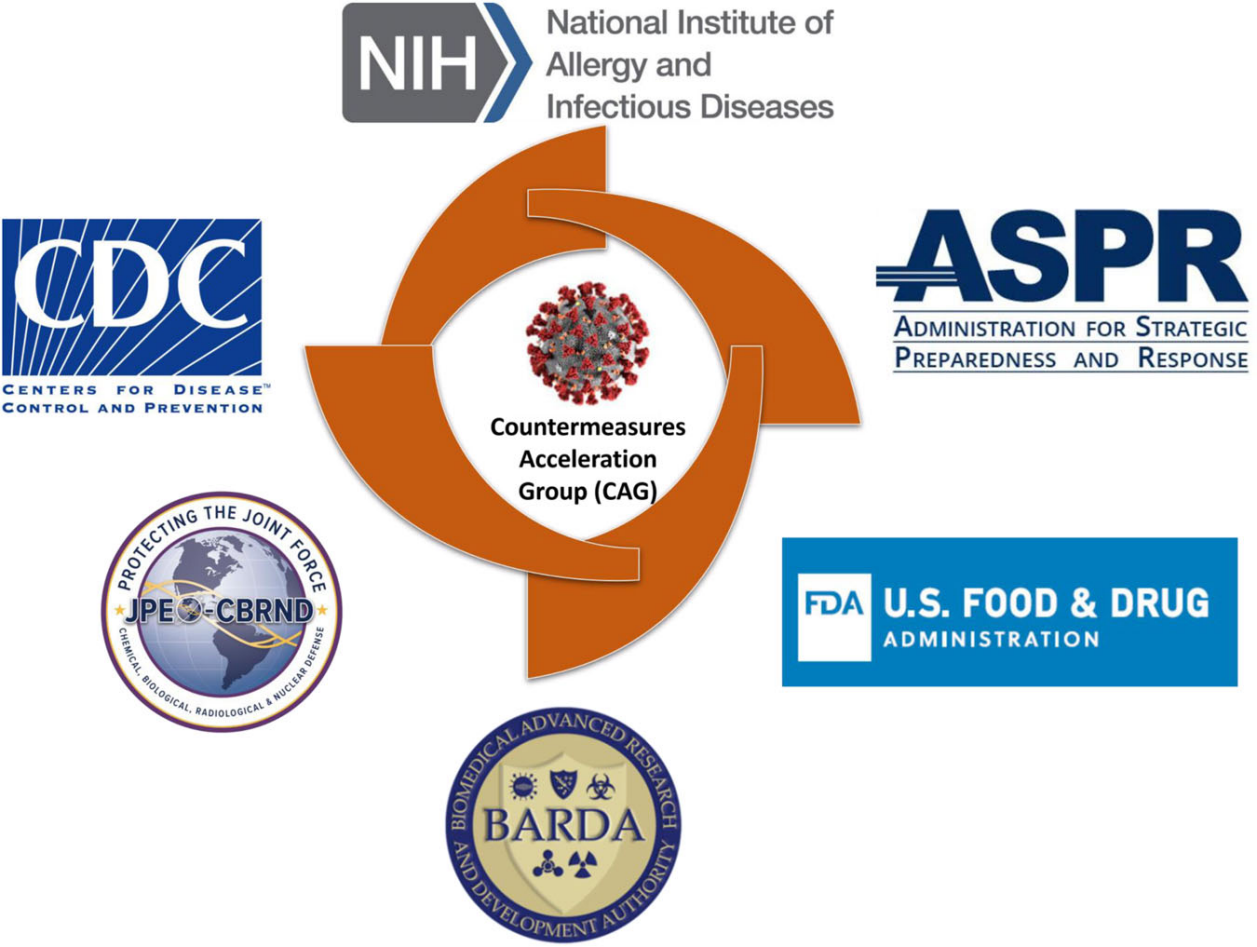
Merging US government centers of excellence for COVID-19. The Countermeasures Acceleration Group, formerly known as Operation Warp Speed, is a partnership between the Departments of Health and Human Services (HHS) and Defense (DoD) with the mission to accelerate the development, manufacturing, and distribution of COVID-19 vaccines, therapeutics, and diagnostics.

The COVID-19 pandemic has shown what is possible when there is an urgent call for collective action. The enormity of the COVID-19 threat required alignment across multiple US agencies to avoid duplication of effort, create opportunities for synergy, and fill identified gaps. Aligned with unity in mission, shared vision, and common goals, this multi-agency effort originated as Operation Warp Speed (OWS) and was later renamed the Countermeasures Acceleration Group (CAG)—(Fig. 2). Similar to the concept of the Manhattan Project, which saw the initial development of nuclear weapons and hastened an end to World War II through scientific collaboration of both government, industry and academia, this effort was a public-private partnership among components of the Departments of Health and Human Services, Defense, and other federal agencies, in engagement with academics and private companies. The OWS/CAG coordinated existing HHS-wide efforts to accelerate the development, manufacturing, and distribution of COVID-19 vaccines, therapeutics, and diagnostics.

The OWS/CAG structure permitted integration of centers of excellence across US government teams and allowed for each entity’s expertize and strengths to be utilized across the framework’s main goals. With decades of experience designing and conducting large-scale Phase 3 clinical trials, and with robust partnerships that were developed for HIV, influenza, and other respiratory pathogens, the NIH/NIAID was able to pivot to the conduct of COVID-19 vaccine efficacy trials. The pivot also leveraged the NIH/NIAID clinical research networks established for HIV and other infectious diseases in the creation of the COVID-19 Prevention Network (CoVPN) to address the pressing need for vaccines and monoclonal antibodies (mAbs) against SARS-CoV-2. The CoVPN is comprised of the NIAID-funded HIV Vaccine Trials Network (HVTN), the HIV Prevention Trials Network (HPTN), the AIDS Clinical Trials Group (ACTG) and the Infectious Disease Clinical Research Consortium (IDCRC). Additionally, the Accelerating COVID-19 Therapeutic Interventions and Vaccines (ACTIV) public-private partnership was formed to help coordinate a research strategy to prioritize and accelerate the development of the most promising treatments and vaccines. Within the NIH and across multiple federal agencies, the coordination of resources, flexibility of established mechanisms, and an all-hands-on-deck approach led to the accelerated review of grants, contracts, agreements, and reporting of results needed to implement these life-saving interventions. A similar culture of urgency and expansion of potential partners, both public and private, would be important to leverage for future HIV vaccine studies.

Harmonization across all Phase 3 vaccine efficacy studies was critical to ensure consistent evaluation of, and comparability across, vaccine products. The NIH/NIAID also helped to establish and convene a single, independent Data and Safety Monitoring Board (DSMB) that provided safety oversight across all US-funded COVID-19 vaccine trials. Further, the use of validated immune assays funded by the NIH allowed for cross-comparison of results across multiple vaccine platforms and enabled the identification of correlates of protection. The Centers for Disease Control (CDC) worked in conjunction with multiple agencies to prepare for and distribute vaccines once authorizedand available for use. Additionally, the Biomedical Advanced Research and Development Authority (BARDA), another arm of the DHHS, was able to utilize the public-private partnership (PPP) model early in the pandemic response by bolstering outreach programs and de-risking product development for industry partners through contracts that offered flexibility and funding support for both research and manufacturing. PPPs have effectively bridged gaps between academia, industry, and funding agencies as public sector commitments are integrated with other key partners experienced in product and business development to improve the health of populations. The engagement of PPPs in vaccine research can be strengthened by flexible governing and funding mechanisms that should evolve to support product development in the PPP model. For example, BARDA utilized the flexibility of the Other Transaction Agreement (OTA) mechanism for flexible portfolio-based funding in which partners jointly decide on candidates to move into and out of the portfolio based on product performance, technical risk, and programmatic need. The US government further assumed financial risk in scaling up manufacturing in parallel, ahead of vaccine efficacy data to support the ongoing clinical development. The advance purchase agreement model was key in minimizing timelines for the development of medical countermeasures. The flexibility afforded by these funding mechanisms was critical during the COVID pandemic, resulting in significant time, effort, and cost savings within the PPPs.

Equally critical was various governments’ engagement with the private sector to advance multiple COVID-19 vaccine candidates. Paramount to the success of these collaborations was open and effective information and data sharing. Merging scientific and clinical trial communities across the public and private sectors allowed for a systematic approach to vaccine development, manufacturing, approval, and distribution. This allowed for a unified mission with shared direction permitting scientific research to be considered in the context of surveillance, manufacturing and scale-up, and public health implementation. Sustained communication and transparency coupled with confidentiality protections and reduction of financial risks allowed for successful engagement with private industry, who are often reluctant to invest in vaccines or other biomedical interventions without adequate management of confidentiality and financial risks. These considerations must be taken into account for HIV vaccine development as governments work towards tangible solutions to incentivize private partners through the implementation of intellectual property and confidentiality protection measures along with funding to de-risk manufacturing investments made during the vaccine development life cycle.

It is also important to address the complex issue of vaccine equity as we apply lessons learned from the COVID-19 response to HIV vaccine research and development. Three key areas can be considered to help pave a way forward towards equitable distribution of vaccines once approved by regulatory authorities. First, producing more vaccines for wider distribution will require persistent government support to help manufacturers expand capacity. Intense competition among pharmaceutical and biotechnology companies has hampered matching COVID-19 vaccine developers to facilities with available capacity. Recently, efforts have been underway to establish collaborative frameworks that would create such opportunities. One example is Pfizer-BioNTech’s use of Sanofi facilities in France to manufacture vaccines and the development of an mRNA manufacturing hub in Africa^29,30^. The establishment of regional manufacturing to address the needs of local countries can be economically feasible and increase return on investment^31^. This results in strengthening vaccine manufacturers through the provision of information programs and professional training on technical improvements, research in vaccine production, and encouraging technology transfer initiatives. The growth and long-term viability of low- and middle-income country manufacturers depends on government commitment, policies supporting capital access, and continuous sponsorship by independent National Regulatory Authorities^32^. While overall economic development has helped to increase public and private access to capital, the need for continued capital investment to ensure successful compliance with manufacturing regulations (e.g., Current Good Manufacturing Practice) and the adoption of new production technology is a particular challenge for manufacturers and important for governments to consider^33^.

Second, efficient and effective vaccine supply chains are needed to scale-up production^34^. Notably, moving from risk management and efficiency-focused supply chain foci towards both resilience-by-design and resilience-by-intervention strategies will be critical to maintaining vaccination targets as supply chain disruptions continue^35^. For example, instead of supply chain risk management strategies that focus on supplier redundancies and flexible warehousing, building resilience into the supply chain management process would allow for use of interchangeable and generic materials, when possible, supplier contracts in different geopolitical regions, and keeping an inventory buffer of critical raw materials. Complimentary to regional manufacturing needs discussed previously, local resourcing and stockpiling of supplies is another critical strategy to address supply chain shortages as these supplies can be manufactured, distributed, and stored locally removing the complexities involved with both importation and exportation of supplies. Perhaps the most notable outcome of the above strategies is the training and recruitment of local talent to manage an efficient vaccine supply chain.

Third, governments must consider policies to support equity. Market forces alone will not suffice to ensure access and deployment in many lower-middle-income countries. Restoring economic growth and progress toward eliminating poverty requires equitable access to vaccines for people in developing countries. This is not only a moral issue requiring altruism. Vaccine inequity is a global health hazard.

When we look to apply lessons learned from COVID-19 to the HIV field, we must first recognize that the HIV vaccine field has long established successful consortia with the, private sector, academic partners, and Governments under shared goals. However, we can continue to build upon these existing partnerships by considering ways to further incentivize companies by de-risking product development through sustained funding and promoting transparent communication early and throughout the product development process. It is important to note that the market incentives for industry participation in HIV vaccine development will need to be significantly higher than for the COVID-19 vaccine effort due to multiple additional factors, such as feasibility, costs, especially for a multidose regimen, regulatory requirements, manufacturing scale-up, and deployment of an efficacious vaccine for high-incidence countries compared with industrialized countries. These challenges will necessitate a strong business case to make this an attractive commercial endeavor.

Another key lesson that can be applied to HIV vaccine research moving forward is the importance of flexibility. The interoperability of government-funded clinical networks, laboratories, and data-sharing platforms is critical to success. Alternative strategies are needed to determine not just what can be done at the level of infrastructure but also with funding and partnership strategies that can be built upon to advance HIV vaccine research and development. Early engagement between the FDA and product developers has been key in the COVID-19 response. Clear guidance from the FDA has also helped to accelerate the timely and thorough review of EUA requests. The ability to have ongoing conversations throughout the development process is vital. As such, updated and clear guidance documents for HIV vaccine developers should be considered moving forward. One of the key considerations moving into the post-pandemic period is how to sustain these labor-intensive and challenging dialogs over the longer term.

All of the above steps are vital, as the COVID-19 experience has shown that the research enterprise can make momentous achievements when resources and innovation are shared between the Government, academia, and biotechnology and pharmaceutical companies. In the end, the “all-of-government” approach, complete with transparency, sustained funding, and the development of public-private partnerships coupled with clear regulatory guidance will be essential in the realization of a safe and effective HIV vaccine.

## Engaging impacted and underserved communities

HIV clinical research has a rich history of engaging communities, especially underserved populations, both domestically and internationally, early on in the research cycle. In fact, in many ways, the HIV research field has refined the concept of community engagement and inclusivity to the point of making it understandable, teachable, and normative^36^. Also, the HIV field has recognized the importance of promptly communicating clinical trial outcomes, whether exciting or disappointing, with clarity, accuracy, and thoughtfulness. However, many lessons may still be gleaned from the COVID-19 experience. In early to mid-2020, the SARS-CoV-2 virus spread quickly throughout the USA, particularly among racial and ethnic minority populations experiencing health inequity^37^. Involving these communities in COVID-19 clinical trials was a top priority for the Government and led to many outreach efforts^38^. For example, the NIH formed the Community Engagement Alliance team to address the needs of the communities most impacted by SAR-CoV-2. At the same time, the CoVPN improved outreach to communities by establishing several Expert Panels comprised of medical, research, and social science experts from different race/ethnic groups, and older adult and veteran populations. In addition, the CoVPN expanded outreach to communities through its Faith Initiative by creating Faith-Leadership groups. The CoVPN Faith Initiative is a national, faith-based program in the US to enhance trust and meaningful engagement in key communities and provide accurate and updated information about COVID-19 and CoVPN clinical trials through the coordinated efforts of seven “faith ambassadors” and more than 30 clergy-consultants from the Black, Latinx and American Indian/Alaska Native communities. These faith leaders are charged with implementing a faith-focused COVID-19 and CoVPN education program that supports the inclusive engagement of members in key communities. Central to all the community outreach was communicating early and continually throughout the research process and managing communications at the local level. Importantly, these outreach groups worked to honor the agency and autonomy of communities as partners in the process, resulting in a more equitable bidirectional relationship. Mistrust of the medical research enterprise, largely due to a history of unethical research, including by the Government of the US^39^, continues to be felt in some communities^36^. Additionally, structural and systemic racism is experienced daily in many communities and can impact access to services and medical care^40^. Maintaining transparency and active communication was critical for building trust in these communities so that individuals were confident in the system and were willing to be active partners in COVID-19 clinical trials. As part of this partnership, the Government also ensured communities most impacted by COVID-19 had access to vaccines as soon as they became available through EUA or licensure.

The HIV vaccine field can now continue to build upon this progress by focusing on continual community engagement and working to dispel mistrust and misinformation. One impact of the COVID-19 pandemic has been significant gains in science literacy among the general public^41,42^. The HIV research field should build on this momentum through public education programs. Importantly though, researchers must heed individuals’ concerns of mistrust and hesitancy and should aim to understand the priorities of communities in order to meet their needs. For many communities, past and/or current experiences with discrimination or neglect may explain the suspicion of research trials and the hesitancy to participate and to receive vaccines^36^. In addition, the history of medical injustices performed on many communities has done much to stoke the fires of mistrust and skepticism, even for ethically performed medical and public health interventions that are proven to be safe and effective^39^. Researchers must continue to strengthen relationships based upon trust or create them where absent. Some approaches to improve this bidirectional relationship include promoting grassroots communication efforts at the local level and expanding community advisory boards to include more members of the community and community advocates, such as faith-based leaders.

The mobilization of resources for the COVID-19 vaccine trials benefitted from the presence of online platforms which could leverage real-world surveillance data to access communities most impacted by COVID-19. This was exemplified by establishing mobile units and satellite sites affiliated with the clinical research sites and located in heavily impacted communities, which helped to enhance visibility and access. These mobilization resources presented their own challenges, including scalability, operability, and deployment. However important lessons can be learned to improve these, including streamlining approvals and onboarding of satellite sites, both domestically and internationally.

Central to reaching impacted communities in the USA was the coupling of real-time data with epidemiologic modeling to evaluate geo-temporal and demographic risks. This was especially important at the start of the COVID-19 pandemic for identifying clinical sites that could enroll the communities most impacted within virus epicenters. This approach enabled faster endpoint accrual and ensured enrollment of diverse populations. The HIV vaccine field is poised to further advance established epidemiologic approaches, including the use of artificial intelligence and machine learning technologies to enhance the modeling of epidemics and the impact of interventions.

The use of COVID-19 registries made epidemiologic data even more useful because it enabled researchers to select interested participants based on location and risk factors. The US Government, with significant help from industry, launched a very successful COVID-19 registry that eventually received entries from over 700,000 individuals. This registry was used by hundreds of clinical sites to access priority populations in an effort to expedite enrollment. The HIV vaccine field is considering this approach with the Red-Ribbon Registry (https://www.helpendhiv.org/red-ribbon-registry/), which would be established as a screening registry to identify potential volunteers to participate in HIV clinical trials conducted by NIAID-funded HIV clinical trial networks. Promoting such registries could greatly accelerate the enrollment of priority populations for future trials.

Lessons can also be learned from adaptations made to clinical trials to reach impacted communities. One distinguishing feature of the COVID-19 studies was the additional virtual component of these studies, which was necessitated by many factors, such as the need for speed and the quarantining of patients with COVID symptoms. In addition to flexible appointment windows and the option for home visits, there were also opportunities for remote visits when no blood work was needed. Studies were also able to facilitate and maintain remote communication through virtual data collection using electronic diaries. However, ‘low-touch’ data collection platforms had to be end-user friendly, and we continue to learn and improve upon the systems that were used for COVID-19. Including elderly populations who are at greater risk for more severe COVID-19 in the studies was important, and additional training in computer literacy to enable electronic reporting must be considered. Since the clinical trials were in part carried out in underserved communities, with disparities in the availability of smartphones and the internet, access to these devices and the internet for all study participants was crucial. Virtual communication and data collection is an important innovation that has the potential to increase recruitment, as well as participant retention.

## Information sharing accelerates progress

Clearly, much scientific progress has been made over the past two years. While the speed of this progress was due in part to adequate funding and the ability to pivot from HIV to COVID-19, the speed was also affected by an unprecedented open-access, highly collaborative, and transparent research approach that enabled rapid information sharing over a broad range of partners, both public and private. Certain scientific journals accelerated the publication of COVID-19 research, allowing breakthrough research to be freely accessible in real time; this approach could greatly accelerate HIV research as well. Also, for data sharing to be more productive, scientists must improve and optimize strategies to integrate data from different sources, as data from different studies is often non-standardized and located throughout many “siloed” repositories. One solution is to follow FAIR (findable, accessible, interoperable, and reusable) guiding principles. Another solution that is currently being pursued is to create central portals for sharing data that have been integrated from multiple sources.

## Opportunities for improvement

It is also important to acknowledge limitations present in the response to the COVID-19 pandemic, for example, by the US Government. The rapid successful development and subsequent approval of multiple vaccines precluded formal evaluation of long-term relative efficacy of different vaccines and combinations, large-scale randomized placebo-controlled trials of new vaccines, and studies in naive populations. Moreover, there was little time to optimize vaccine doses and schedules, and therefore challenges remain in determining ideal vaccine dosing intervals and how to maximize durability. Over time the relevance of vaccine efficacy in naive populations waned as did the importance of vaccine efficacy against earlier circulating viruses in this dynamic pandemic. More studies are needed that include higher proportions of special populations, such as people living with HIV/AIDS, organ transplant recipients, cancer patients on chemotherapy, pregnant women, and children. The timely inclusion of some of these more vulnerable populations could have better-informed vaccine strategies for wider benefit. Learning from these limitations will strengthen strategies in vaccine development for HIV and future pandemics.

## Conclusion

Science met the challenges of the COVID-19 pandemic with unprecedented speed and accuracy with the development of safe and effective vaccines. Now is the time to harness the collective momentum from COVID-19 for HIV. Throughout all the approaches described here, several overarching themes emerge: flexibility is critical for responding to an ever-changing pandemic; inclusion improves equity in access to vaccines, therapeutics, and services for communities most impacted by a pandemic (although international access remains a continuing challenge), enables a diversity of thought, and improves decision-making; transparency is vital to accelerate science and promote trust; and continued investment in basic research supports the search for an efficacious HIV vaccine. By learning from these important lessons, the scientific community can capitalize on the momentum of the COVID-19 experience to re-energize HIV vaccine development.
